# Establishment of CRISPR-Cas9-Mediated Gene Editing in the Swimming Crab *Portunus trituberculatus*

**DOI:** 10.3390/molecules31020285

**Published:** 2026-01-13

**Authors:** Xiaopeng Wang, Xuhao Chen, Yueyue Zhou, Yun Zhao, Ce Shi, Ronghua Li, Lei Liu, Changkao Mu, Weiwei Song, Chunlin Wang

**Affiliations:** 1 Marine Economic Research Center, Donghai Academy, Ningbo University, Ningbo 315000, China; wangxiaopeng@nbu.edu.cn (X.W.);; 2 Key Laboratory of Green Mariculture (Co-Construction by Ministry and Province), Ministry of Agriculture and Rural, Ningbo 315000, China; 3 Key Laboratory of Aquacultral Biotechnology, Chinese Ministry of Education, Ningbo University, Ningbo 315000, China; 4 Collaborative Innovation Center for Zhejiang Marine High-Efficiency and Healthy Aquaculture, Ningbo 315000, China

**Keywords:** *Portunus trituberculatus*, CRISPR-Cas9, gene editing, electroporation, *mstn*

## Abstract

*Portunus trituberculatus* is an economically important marine crustacean in East Asia’s aquaculture industry. Nevertheless, precise genome modification has not yet been established. In this study, we evaluated the applicability of the CRISPR-Cas9 gene editing system in *P. trituberculatus* using electroporation for efficient delivery of the Cas9-sgRNA complex into zygotes. We systematically investigated electroporation parameters, including buffer composition, voltage, capacitance, and pulse times. Our results showed that artificial seawater was a superior buffer to phosphate-buffered saline (PBS) and identified an effective electroporation condition of 600 V, 1 μF capacitance, and two pulses, resulting in approximately 72.7% fluorescent zygotes. Under these electroporated conditions, we detected gene indels and putative insertion events at the targeted locus of myostatin (*mstn*) gene. These results demonstrate the feasibility of Cas9-based genome editing in *P. trituberculatus* and provide a proof-of-concept for functional genomics studies and future genetic improvement of this species.

## 1. Introduction

*Portunus trituberculatus*, commonly known as the swimming crab, is a significant marine crustacean species in China, belonging to the Crustacea subphylum, Decapoda class, and Portunidae family. Its large body size, rapid growth, and distinctive nutritional and flavor qualities make it an important economic species for marine aquaculture in the region [1,2]. Based on the China Fishery Statistical Yearbook, the aquaculture production of *P. trituberculatus* in China has reached over one million tons in recent years. Along with customers’ increasing demand for excellent protein from the swimming crabs, aquaculture production must ramp up consistently. Currently, the aquaculture industry primarily relies on conventional selective breeding [3,4] methods to select the superior germplasm and advanced factory farming techniques [5,6] to enhance the aquaculture animals’ yield. However, due to the unique physiological [7] and genetic traits [8] of *P. trituberculatus*, its yield has yet to be significantly improved. Recently, CRISPR-Cas9 technology has emerged as a promising tool for precise genome editing across species, enabling targeted genetic improvements that can be inherited by subsequent generations. This technology may offer a shortcut for breeding *P. trituberculatus*.

With the remarkable specificity, eco-friendliness, and adaptable design [9,10], CRISPR-Cas9 has emerged as a prominent technique. Originally identified as a bacterial adaptive immune system, the CRISPR-Cas9 system [11] comprises the Cas9 nuclease, a CRISPR RNA (crRNA) array, and a trans-activating CRISPR RNA (tracrRNA). By combining the crRNA and tracrRNA, a chimeric single-guide RNA (sgRNA) is generated, enabling Cas9 to introduce DNA double-strand breaks (DSBs) at nearly any desired target site [12]. DSBs are among the most dangerous lesions for DNA, resulting in cell death if ineffectively repaired [13]. DSBs are primarily repaired through two key pathways: the template-dependent homologous recombination (HR) and the template-independent classical non-homologous end joining (NHEJ). HR, confined to the S/G2 phases of the cell cycle, is less frequent compared to NHEJ [14]. Although HR generally happens at lower frequencies than NHEJ, it enables precise and specific modifications at a target locus when an external repair template is available. In contrast, DSBs repaired via NHEJ often result in insertion/deletion (indel) mutations, which leave genetic scars [15]. Among crustaceans, the successful application of CRISPR-Cas9 has been reported in the species of the amphipod *Parhyale hawaiensis* [16], the decapod *Exopalaemon carinicauda* [17,18], *Neocaridina heteropoda* [19], *Macrobrachium nipponense* [20], *M. rosenbergii* [21], and the freshwater crab *Eriocheir sinensis* [19]. Yet no study in the commercially significant swimming crab *P. trituberculatus* has been reported.

Similar to other reported crustaceans, the zygotes of *P. trituberculatus* go through oviparous embryonic development during their early stages [22,23]. Unfavorably, the single-cell stage of the zygotes only holds a relatively short time window, which is less than 6 h. Meanwhile, the oviposition is usually in the range of 10^5^–10^6^ at a time, supplying us with adequate experimental materials. Given the limited time frame for the development of single-cell zygotes and the vast number of ovipositions, utilizing a high-throughput approach for large-scale experimentation may increase the chances of success in gene editing. To address this challenge, we propose a high-throughput electroporation delivery method, aiming to improve gene editing efficiency. An electroporator is a highly effective apparatus that utilizes short yet powerful electric pulses to temporarily enhance the permeability of cell membranes, allowing exogenous substances (e.g., DNA, RNA, proteins, etc.) to enter the cell [24,25,26,27]. Electroporation is widely employed in various fields, ranging from gene transfection and cell therapy to drug delivery and cell transformation [28,29]. Gene editing in crustacean zygotes has primarily been achieved through microinjection systems [17,19,20,21]. However, microinjection is labor-intensive, requires advanced technical expertise, and can only inject one cell at a time [30]. Therefore, a high-throughput electroporation method capable of processing multiple cells simultaneously has yet to be developed.

In this study, we developed a high-throughput and efficient electroporation delivery method in *P. trituberculatus*. Targeting the myostatin gene (*mstn*), we explored the effects of electroporation parameters on the zygotes, including voltage, capacitance, pulse times, and electroporation buffer. Eventually, we successfully realized the mutation of the targeting locus. This research not only extends gene editing techniques for crustaceans but also lays experimental groundwork for future enhancement of germplasm resources of *P. trituberculatus*. This will lead to an increase in aquaculture production and economic benefits, as well as the exploration of its gene function and biological processes.

## 2. Materials and Methods

### 2.1. Experimental Animals and Bacteria

The nearly spawning crabs were purchased from Qixin farm in Ningbo and put in the mid-trial base of the Meishan Campus of Ningbo University for temporary rearing. Fresh razor clam bait was fed regularly at 10% of the crab’s body weight to ensure its nutrient intake; the water temperature was monitored at about 25 °C, salinity at about 25‰, and ammonia nitrogen content in the water (less than 0.2 mg/L); and the water was changed every other day. Underwater cameras were used to monitor the daily activity behavior of the crabs until they spawned. *Escherichia coli* DH5a and DE3 were used as molecular cloning and heterologous expression strains, respectively. *Escherichia coli* was cultured in LB liquid medium at 37 °C and 200 rpm in a shaker or on LB solid plates in the static incubator. Appropriate antibiotics were added to the culture of the engineered strains, such as ampicillin (final concentration of 100 mg/L) or kanamycin (50 mg/L). Plasmids and bacteria information were attached in Table 1. All animal handling and experimental protocols strictly adhered to the principles set forth in the Regulations for the Administration of Laboratory Animals of Zhejiang Province (Government Order No. 263, 2009) and the national Guidelines for the Humane Treatment of Laboratory Animals of China. The Ningbo University Ethics Committee confirmed that no additional ethics approval was required for this study.

### 2.2. Mstn Gene Sequence Validation and sgRNA Design

The target gene was myostatin (*mstn*, GenBank accession No.: HQ693759.2). Specific primers were designed (Table 2) to amplify the target gene fragment. The PCR procedure was performed using LA taq PCR enzyme (Takara, RR02AG, Dalian, China) under the kit instructions. After purification and target band recovery, the DNA product was ligated into the pEASY^®^-Blunt Cloning Kit (Transgene, CB101-01, Beijing, China) and sequenced using M13 universal primers. Then, the full-length sequence was spliced using the Seqman software (DNASTAR Lasergene version 7.1, DNASTAR Inc., Madison, WI, USA). Based on the sequence information obtained through second-generation sequencing, multiple groups of gRNAs were screened, referring to the screening criteria in the previous report [33], with a 20 nt spacer sequence followed by the 5′-NGG (PAM sequence) [34], and finally, four groups of sgRNAs were identified. The corresponding specific amplification primers were designed based on the sgRNA information (Table 2) and sent to BGI Company for synthesis.

### 2.3. Plasmid and Engineering Strain Construction

The Cas9-eGFP expression vector PMJ922 was purchased from Addgene (#78312, Martin Jinek laboratory, Cambridge, MA, USA) [31], and the sgRNA expression cassette vector pgRNA was from our lab stock [32]. The construction process of the donor vector of pDonor-CMV-mcherry, which can be used for phenotypic verification after the insertion of the exogenous gene, is as follows (Figure S1): Upstream sequence of *mstn* gene was obtained by regular PCR using genomic DNA as template, while restriction enzyme recognition sites of HindⅢ and XbaI were incorporated at both termini of the sequence to enable efficient downstream vector construction. Downstream sequence of the *mstn* gene was prepared likewise, with the addition of XhoI and KpnI enzymatic sites. The CMV-mcherry expression cassette [35] was constructed by an artificial synthetic method with the addition of restriction enzyme sites of XhoI and XbaI. Then, the upstream and downstream donor fragments and the CMV-mcherry expression cassette were double-enzyme digested using corresponding enzymes, followed by the overnight ligation using T4 ligase (Takara, 2011A), with the pUC57 plasmid backbone, double-enzyme digested by HindⅢ and KpnI. After transformation and sequencing using M13 primers (Table 2), engineering bacteria strains containing pgRNA, PMJ922 and pDonor-CMV-mcherry were constructed completely, respectively. The donor plasmid of pDonor-CMV-mcherry was extracted and used to transform zygotes of *P. trituberculatus*. The pgRNA was used for sgRNA preparation. The PMJ922 was used for Cas9-eGFP protein purification.

### 2.4. Cas9-eGFP Protein Purification, sgRNA Preparation, and In Vitro Cleavage Detection

Cas9-eGFP purification procedure was carried out following the reported protocol [31]. Briefly, the overnight culture of Cas9-eGFP-expressing strain was transferred to 1 L of LB medium, and incubation was continued at 37 °C, 200 rpm, until OD600 = 0.5. Then, IPTG was added at a final concentration of 500 μM and induced for 18 h in a shaker for 20 h at 18 °C, 200 rpm. Afterward, the precipitated bacteria were collected and resuspended in an equilibration buffer (20 mM imidazole, 300 mM NaCl, 50 mM Na_3_PO_4_), and then the cells were fragmented using an ultrasonic disruptor (Scientz, Ningbo, China). The supernatant was collected by centrifugation (8000 rpm, 30 min, 4 °C) and then purified. The lysate was filtered through 0.45 μm filters, then applied to a His60 Ni Gravity Column Purification Kit (Clontech, 635658, Takara Bio Inc., Shiga, Japan), washed, and then eluted with a gradient of imidazole. TEV protease (Solarbio, P2060, Beijing Solarbio Science & Technology Co., Ltd., Beijing, China) was added, and the sample was dialyzed overnight into TEV buffer (500 mM NaCl, 50 mM Hepes, pH 7, 5 mM MgCl_2_, 2 mM DTT). After dialysis, TEV cleavage was confirmed by SDS-PAGE, and the sample was concentrated to 500 μL using ultrafiltration centrifugal tube (Pall, MAP100C36, Port Washington, NY, USA) and resolved in SEC buffer (20 mM HEPES-KOH, pH 7.5, 500 mM KCl, 1 mM DTT). Proteins were analyzed by SDS-PAGE and either used directly for biochemical assays or frozen at −80 °C for storage.

The sgRNA expression plasmid of pgRNA was used as a template, and the corresponding sgRNA primers (Table 2) were applied to amplify the T7-sgRNA expression cassette fragment using 2×Estaq PCR enzyme (CWBIO, CW0690, Beijing ComWin Biotech Co., Ltd., Beijing, China). Afterward, in vitro transcription was carried out to obtain the corresponding sgRNA template, using T7 in vitro transcription kit (Transgene, JT101, Beijing, China). The transcribed sgRNA was purified and examined according to the kit instructions. The in vitro cleavage effect was detected using the Cas9 protein (GenScript, Z03388, Nanjing, China), sgRNA, and *mstn* template prepared above. The cleavage reaction used 100 ng of transcribed sgRNA, 160 ng of target DNA, and 50 ng of purified Cas9 protein, referring to the protocol [36]. Reactions were terminated by adding 1 μL proteinase K (20 μg/μL), incubating for 20 min at 50 °C, and the reaction products were examined by 1% agarose electrophoresis.

### 2.5. Electroporation Parameters and Fluorescence Observation

Preliminary single-factor optimization experiments were conducted to evaluate the effects of electroporation conditions on zygote survival. Zygotes were collected from newly spawned, egg-carrying crabs, washed with sterile artificial seawater (AS), and dispersed into individual eggs. Approximately 200 fertilized eggs were transferred into a 0.2 cm gap electroporation cuvette (Bio-Rad, Hercules, CA, USA) containing 60 µL of buffer (with CRISPR components if applicable) and gently mixed. Electroporation was performed using an exponentially decaying pulse generator (Scientz-2C, China). Constant parameters in all experiments included resistance (400 Ω), time constant (~10 ms), and sample volume (~200–300 zygotes in 60 µL buffer). Other device-specific parameters were not directly measurable.

Single-factor experiments varied one parameter at a time while keeping others constant: (i) Buffer: AS or PBS; voltage 500 V, capacitance 31 µF, resistance 400 Ω, pulse 1. (ii) Voltage: 400–600 V; buffer AS, capacitance 31 µF, resistance 400 Ω, pulse 1. (iii) Capacitance: 1, 6, 25, 31, 100 µF; buffer AS, voltage 500 V, resistance 400 Ω, pulse 1. (iv) Pulse number: 1–3; buffer AS, voltage 450/500/600 V, capacitance 31 µF, resistance 400 Ω. Each experiment included three biological replicates (fertilized eggs from one female crab each). After electroporation, zygotes were incubated in 90 mm Petri dishes with 30 mL sterile seawater at 25 °C for 24 h. Survival was assessed microscopically based on morphology, cleavage, and cytoplasmic clarity.

Based on the results of the single-factor optimization experiments, electroporation parameters within the tolerable range were combined to evaluate Cas9-eGFP protein delivery into zygotes, rather than to estimate genome-editing efficiency. Voltage was set at 400, 500, or 600 V, capacitance at 1 or 6 µF, and the number of pulses was varied from 1 to 3, while all other electroporation parameters were kept constant as described above. Zygote survival was assessed using the previously described criteria. Cas9-eGFP protein uptake was evaluated qualitatively and semi-quantitatively by counting fluorescent-positive zygotes under a fluorescence microscope 24 h post-electroporation. All experiments were conducted with three independent biological replicates (*n* = 3), with each replicate comprising fertilized eggs obtained from a single female crab. Fluorescence observations were performed using a Nikon Eclipse Ti inverted fluorescence microscope (Nikon, Chiyoda-ku, Japan). Images were acquired and processed using NIS-Elements D software (version 4.60.00).

### 2.6. Ribonucleoprotein (RNP) Electroporation into Zygotes of P. trituberculatus and Artificial Incubation

For RNP complex assembly, Cas9 protein (12 μg) was mixed with sgRNA (18 μg), with or without donor plasmid (12 μg), and incubated at 37 °C for 5 min as previously described (Burger et al., 2016) [31]. The preassembled RNP complexes were introduced into single-cell zygotes of *P. trituberculatus* via electroporation using an exponentially decaying pulse system (Scientz-2C, China). Based on prior parameter optimization experiments, electroporation was performed at 600 V, 1 μF capacitance, and 400 Ω resistance, with two pulses, using a 0.2 cm gap cuvette containing approximately 200 zygotes suspended in 60 μL electroporation buffer. All experimental groups were conducted with ten independent replicates, comprising a total of approximately 2000 zygotes per group. Following electroporation, zygotes were transferred to an artificial incubation system (Figure S2) and cultured according to procedures described in our previous study (Patent Application No. 2021220055713.6). Culture conditions were maintained with daily water renewal, pH 8.0–8.5, temperature 20–25 °C, salinity 20–25‰, dissolved oxygen ~8 mg/L, and natural indoor light. Zygote survival was assessed microscopically at 24 h and 96 h post-electroporation. Due to progressive mortality during incubation, all surviving larvae at day 13 were pooled within each group, and hatching outcome was expressed as the total number of surviving larvae and the corresponding percentage relative to the initial number of treated zygotes.

### 2.7. Genomic DNA Extraction and PCR-Based Analysis of Genome Editing Events

To maximize sample utilization (Table 3), all surviving larvae from electroporated groups and a subset of control larvae were collected individually for genomic DNA extraction using a Tissue DNA Kit (Omega Bio-Tek, D3396, Norcross, GA, USA). PCR was performed with MSTN F2 (from the endogenous mstn sequence) and mCherry R (from the donor plasmid) to detect donor-related sequences. Amplicons were checked by agarose gel electrophoresis, purified, and Sanger sequenced using MSTN F1 (upstream of the putative insertion). Primer sequences and expected amplicons are listed in Table 2. Sequencing results were aligned using SnapGene version 6.0.2 (GSL Biotech LLC, Chicago, IL, USA). This PCR strategy provides preliminary evidence for donor-derived sequences but does not rule out residual plasmid or fully confirm precise HDR-mediated insertion; thus, results are interpreted as proof-of-concept pending further validation with junction-spanning primers and additional assays.

### 2.8. Statistical Analysis

The 24 h survival rates of zygotes under different electroporation parameters were used to guide the selection of experimental conditions. For the single-factor parameter optimization experiments, each condition was repeated in three independent replicates, using zygotes derived from separate female crabs. For the RNP electroporation experiments, each experimental group was conducted with ten replicates, each consisting of approximately 200 zygotes. Due to progressive mortality during incubation, all surviving larvae at day 13 were pooled within each group for molecular analysis (see Section 2.7). Survival rates at 24 h and 96 h were calculated for each replicate and are reported as mean ± SD. Statistical comparisons among groups at early time points (24 and 96 h) were performed using one-way ANOVA followed by Tukey’s post hoc test, with *p* < 0.05 considered significant. Hatching outcomes at day 13 are presented as descriptive totals and percentages relative to the initial number of treated zygotes; due to low survival, no inferential statistics were applied to these data. Graphs were generated using GraphPad Prism software version 8.3.0 (GraphPad Software, San Diego, CA, USA), and layouts were adjusted in CorelDRAW Graphics Suite 2020 (Corel Corporation, Ottawa, ON, Canada).

## 3. Results

### 3.1. Guide RNAs Exhibited Diverse Cleavage Efficiencies In Vitro

**Figure 1:**
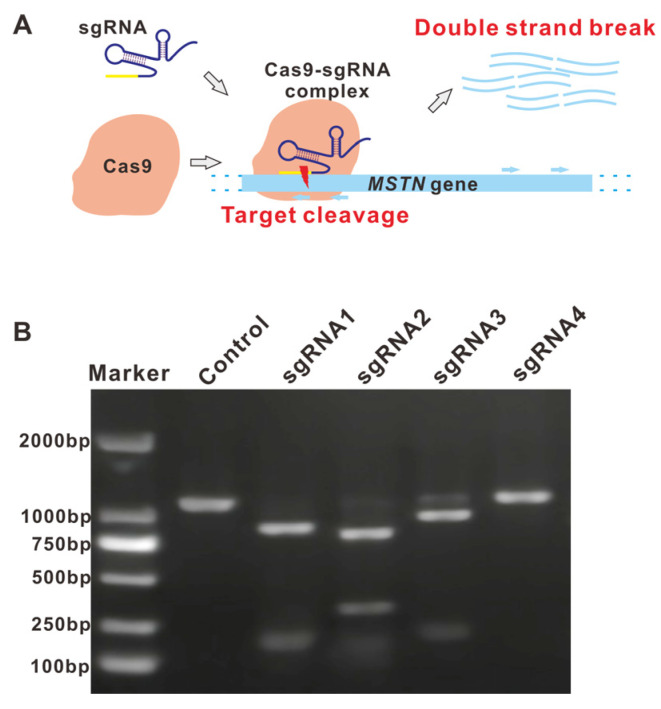
Targeted cleavage effect of Cas9-sgRNA. (**A**) Mechanism of Cas-sgRNA targeted cleavage effect. Light blue arrows indicate position of sgRNAs; Dotted lines indicates double strand DNA. (**B**) In vitro cleavage effects of various sgRNAs. Theoretical cleavage sizes were calculated based on genomic sequence: Control, 1146 bp; sgRNA1, 889 bp + 257 bp; sgRNA2, 853 bp + 293 bp; sgRNA3, 786 bp + 360 bp; sgRNA4, 832 bp + 314 bp.

To determine the cutting efficiency of different cleavage sites, we performed the in vitro cleavage assay using 4 sgRNAs, two targeting the plus (+) and minus (−) strands of the ORF region of the *mstn* gene, respectively. As can be seen in Figure 1A, the Cas9 protein was incorporated with sgRNA, forming an RNP complex that performed targeted cleavage at the locus site, guided by the sgRNA. As shown by the gel electrophoresis (Figure 1B), the cleavage ability of different sites exhibited significant differences, which can be distinguished from the residual template DNA. Among them, the sgRNA4 group did not show cleavage, and the other three groups were cleaved. The size of the PCR product bands obtained after cleavage was consistent with the expected size (see figure legend for detailed sizes). Consequently, sgRNA1 and sgRNA2 were selected as the targets for subsequent experiments.

### 3.2. Electroporation Conditions Affected the Survival Rates of Zygotes

**Figure 2:**
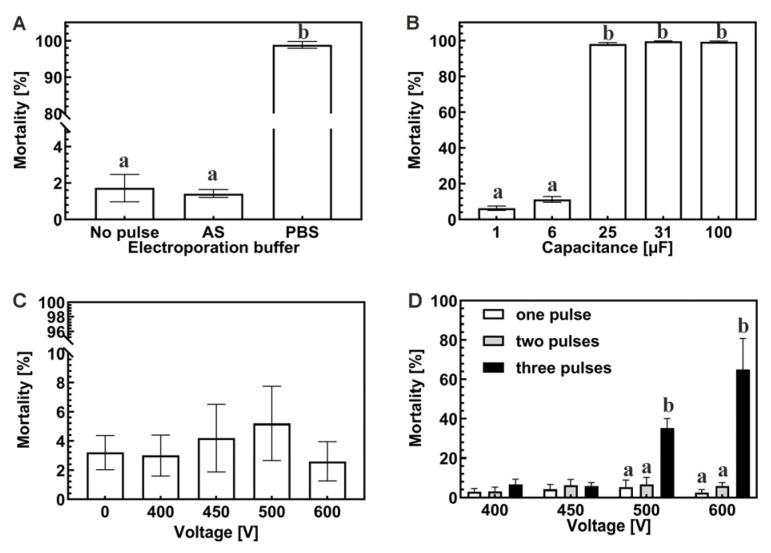
Comparison of zygote mortality rate under different electroporation conditions. (**A**) Electroporation buffer: Artificial seawater (AS); Phosphate-buffered saline (PBS). (**B**) Capacitance: 1–100 μF. (**C**) Voltage: 0–600 V. (**D**) Different voltage and pulse time combinations. Different superscript letters indicate significant differences among groups.

Electroporation buffer. The appropriate ion concentration and types in electroporation buffer are essential for maintaining the physiological properties of zygotes under electric pulses. Two types of buffers were investigated to select the appropriate buffering condition. As can be seen from Figure 2A, the commonly used phosphate-buffered saline (PBS) solution significantly negatively affected the 24 h survival of zygotes, as the mortality reached 98%. In contrast, the AS provided a better environment for the zygotes, exhibiting a lower lethality of 2%, which is comparable to the control group without a pulse (*p* > 0.05). Therefore, AS was selected for further experiments.

Capacitance. Capacitance refers to the ability of a capacitor to store charge and plays a vital role in electroporation. To determine the appropriate capacitance parameters, we examined the survival rate of zygotes under different capacitance conditions. The results are shown in Figure 2B. As can be seen, the lethality of zygotes with capacitance values of 25 μF, 31 μF, and 100 μF exceeded 98% (*p* > 0.05), whereas the lethality of 1 μF and 6 μF was 6.33% and 11.33% (*p* > 0.05), respectively. In summary, higher capacitance causes more damage to the zygotes, and capacitance no higher than 6 μF was tolerable.

Voltage. Voltage is one of the critical factors affecting the efficiency of electroporation. To balance between the efficiency of electroporation and the survival rate of the zygotes after the pulse, we examined the survival rate of the zygotes under different voltage parameters. From Figure 2C, the results showed that the 24 h mortality rate of zygotes ranged from 2.60 to 5.20% as the voltage increased from 400 V to 600 V, with no significance from the control group (*p* > 0.05). It indicates that this voltage value is within the tolerance range of zygotes. In addition, when the voltage was higher than 600 V, arcing occurred in the electroporation cuvette, which limited further optimization. Therefore, the voltage was set at or below 600 V.

Pulse times. Under the tolerable voltage, multiple pulses can theoretically produce better perforation effects, thus improving the electroporation efficiency. To investigate the effect of pulse times on zygotes, up to three pulses with voltages lower than 600 V were performed. As can be seen in Figure 2D, when the voltage was lower than 450 V, three pulses had no significant effect on the 24 h mortality of zygotes (*p* > 0.05); when the voltage exceeded 500 V, the mortality rate of zygotes increased significantly with the number of pulses, reaching up to 35.4% (500 V) and 65.0% (600 V) at 3 pulses, respectively. Therefore, the pulse times were set at or below 2 in the following experiment.

### 3.3. Cas9-eGFP Can Be Imported into Zygotes Under Proper Electroporation Conditions

**Figure 3:**
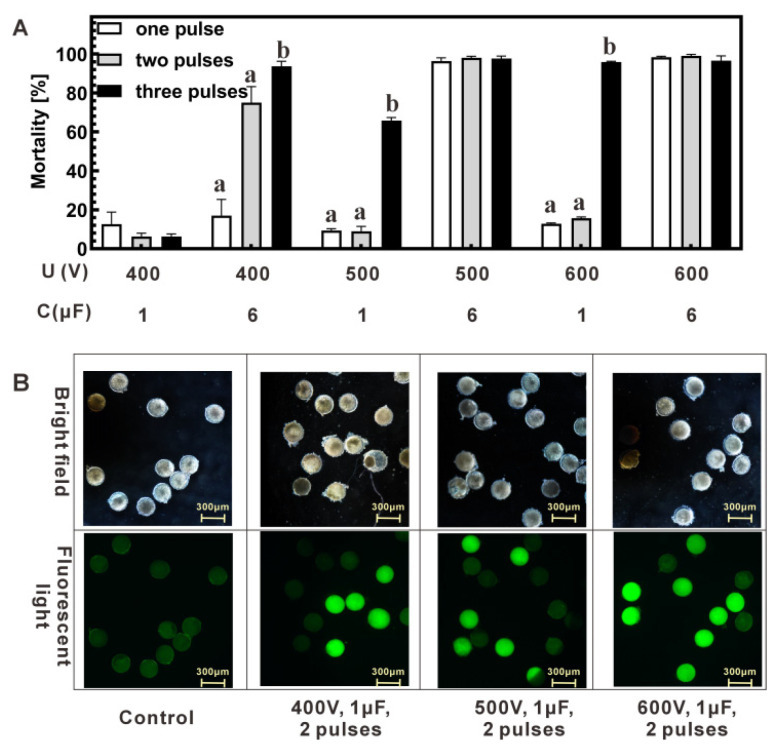
Comparison of mortality rates and fluorescence in zygotes following optimized parameters. (**A**) Evaluation of parameter combinations: Voltage, capacitance and pulse times in electroporation. (**B**) Microscopic observation of electroporated zygotes under a 10× objective. The upper panel displays bright-field images, while the lower panel shows GFP fluorescence. Green fluorescent zygotes indicate potential importation of GFP. Different superscript letters indicate significant differences among groups.

Following optimization of buffer, voltage, capacitance, and pulse number, various parameter combinations were tested for Cas9-eGFP import. Voltage ranged 400–600 V, capacitance was 1 or 6 μF, and 1–3 pulses were applied. Mortality increased with higher capacitance at the same voltage (Figure 3A). At 1 μF, two pulses resulted in 24 h mortality of 6–15% (400–600 V), whereas 6 μF caused higher mortality (38–>95%, depending on voltage), confirming that capacitance ≤ 6 μF was tolerable. Cas9-eGFP import efficiency under 1 μF was assessed by fluorescence microscopy. In two-pulse groups, the percentage of fluorescent zygotes increased with voltage: 400 V, 24.7% ± 5.0%; 500 V, 38.4% ± 2.0%; 600 V, 72.7% ± 10.0% (mean ± SD, n = 3 biological replicates) (Figure 3B and Figure S3). Three pulses at 500 V also yielded high import (60.4% ± 6.0%), but mortality was substantially higher (65.8% ± 1.6%; Figure 3A) compared with 600 V, 2 pulses (15.6% ± 0.8%). Considering both mortality and fluorescence, 600 V with 2 pulses was selected as the optimal condition for RNP introduction.

### 3.4. Genotypic Differences Exist in Zygotes After RNP Introduction

The RNP groups, with or without donor DNA, were used to evaluate the feasibility of electroporation-mediated genome editing. Zygotes were allowed to develop to the larval stage for both genotypic and phenotypic analyses. As shown in Table 3, neither the AS-only control group nor the Cas9-eGFP-only control group showed significant effects on short-term (24 h, *p* > 0.05) or long-term (13 d, *p* > 0.05) development, with final hatching rates around 30%. In contrast, all electroporated experimental groups exhibited very low larval survival, with less than 2% surviving at day 13. Among the four experimental groups, two were designed for mstn knockout (Cas9+sgRNA1 and Cas9+sgRNA2), and two for gene insertion (Cas9+sgRNA1+Donor and Cas9+sgRNA2+Donor) using a donor plasmid carrying the CMV-mCherry cassette. Due to the low survival, fewer than four larvae survived in each group, and a total of 11 larvae were collected for molecular analysis.

**Figure 4:**
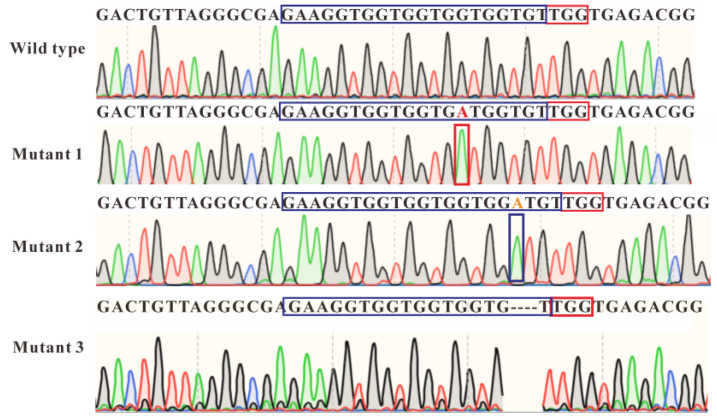
Sanger sequencing results of surviving larvae from the control and Cas9+sgRNA1 groups. Wild-type sequence from no-pulse group; Mutant 1, red capital “A” letter represents a single-base substitution; Mutant 2, orange capital “A” letter represents a single-base insertion; Mutant 3, 3-base “GTG” in sgRNA sequence was deleted. Dashed lines indicates 3-base deletion in target locus. Different colors in the Sanger sequencing chromatogram represent different nucleotide signals: A (green), T (red), G (black), and C (blue). Blue box indicates 20 nt sgRNA sequence; red box indicates 3 nt PAM motif.

Genomic DNA from each larva was extracted, PCR amplified, and Sanger sequenced. In the Cas9+sgRNA1 group, three potential mutants were identified: Mutant 1 carried a single-base substitution (T → A) at the 14th position of the sgRNA target, Mutant 2 had a single-base insertion (A) at the 17th position, and Mutant 3 showed a three-base deletion (GTG) from positions 17–19. All mutations occurred near the upstream region of the PAM (TGG motif), consistent with Cas9 editing activity. In the Cas9+sgRNA2+Donor group, PCR and sequencing suggested the presence of the CMV-mCherry cassette in the surviving larvae. However, because the donor plasmid contains homology arms, residual plasmid cannot be completely excluded. Therefore, these results are interpreted as putative insertion events rather than confirmed precise HDR-mediated genomic integration. Overall, these results demonstrate the proof-of-concept that CRISPR components can be delivered into *P. trituberculatus* zygotes via electroporation and induce genomic modifications, while larger-scale studies are required to validate efficiency and reproducibility (Figure 4).

## 4. Discussion

*P. trituberculatus* is an economically essential crab species to which gene editing technology has not yet been successfully applied. The major hurdle lies in the double membrane barrier formed by the egg and cell membranes of zygotes, posing an obstacle to entering exogenous gene editing components [37]. In this study, we optimized electroporation parameters to enable RNP delivery into single-cell zygotes as a proof-of-concept. Limited indels and putative insertions in the mstn gene were detected, representing the first report of genome editing in this non-model organism.

Electroporation devices can generate a transient, high-intensity electric field, which leads to temporary pores on the cell surface, providing an opportunity for exogenous molecules to enter the cell [38]. However, appropriate appliance parameter settings are needed to ensure the generation of the perforation effect and the survival of treated cells [39]. The apparatus’s voltage and capacitance parameters affect the pulse’s intensity, determining how much damage can be inflicted on the cells [28,40]. Excessively high electric fields can alter cell morphology, increasing the number of cell membrane pores. After the withdrawal of the applied electric field, the pores on the cell membrane will not be able to close by themselves, leading to an increase in the amount of cell death [41]; too low of an electric field will result in the cell membrane not easily producing reversible pores, and the exogenous DNA molecules cannot enter the cell, leading to low transformation efficiency. In our study, zygotes tolerated voltages up to 600 V (Figure 2C) and capacitance up to 6 μF (Figure 2B), values that differ from those used in bacteria [42,43], mouse zygotes [24], and fish cells [44,45].

Electroporation buffers affect the efficiency of transformation through the ion environment, including types and concentrations [46]. Electroporation buffer can enter the cells through the nanopores on the cell membrane caused by the electric pulse, and the cells are more sensitive to the osmolarity and composition of the incoming buffer [47,48]. It has been reported that the cell survival rate is higher when the cells are incubated with a buffer similar to that of the cytoplasm [49,50]. The ionic composition and osmolarity of the electroporation buffer also influence cell survival and delivery efficiency [46,47,48,49,50]. We found that artificial seawater (AS) supported high survival, whereas PBS caused >95% lethality (Figure 2A), likely due to osmotic and ionic differences. The optimization of the above parameters laid the foundation for successful editing.

The effectiveness of gene editing also depends on the biological function of imported elements [30]. There exist several advantages over the DNA or mRNA form of Cas9-sgRNA. Specifically, the mRNA form of Cas9 needs to rely on host cells to complete the translation process, and the single-stranded form of Cas9-mRNA is not convenient for storage and requires specific modification to prevent degradation in vivo [51]; The plasmid DNA requires stable replication of the exogenous DNA vector [52] in the host, with efficient screening markers such as antibiotics and fluorescent tags. Furthermore, Cas9 genes need to be codon-optimized for better adaptation to host genetic code preferences, and matching promoters, terminators, and other genetic structures are also required. These necessities still need to be well addressed in non-model organisms like *P. trituberculatus*.

Upon encountering target DNA, the Cas9-sgRNA RNP can induce double-strand breaks. In the presence of donor DNA, HDR may mediate insertions, whereas NHEJ can create indels in the absence of donor DNA [53]. HDR events are generally less frequent than NHEJ. Consistent with our observations (Table 3), surviving larvae exhibited both indels and putative insertions. In the sgRNA2+Cas9+Donor group, sequencing indicated the presence of the CMV-mCherry cassette (Figure S4), although no fluorescence was detected, possibly due to developmental stage or heterologous expression limitations. Although the *mcherry* gene has been codon-optimized according to the host preference, the limited research on genetic elements suitable for *P. trituberculatus* may also need further exploration. In our forthcoming research, we plan to enhance the efficiency of insertional mutations through various strategies. These include adjusting the length of the donor DNA, fusing the Cas9 protein with a single-stranded DNA (ssDNA) donor, or exploring alternative Cas proteins, such as xCas9, to improve the efficiency of insertional mutations.

On the other hand, NHEJ repair would rarely result in point mutagenesis, as it usually creates indels. The point mutation single-base substitutions (T → A) observed in the Cas9+sgRNA1 group, occurring near the PAM sequence, may be caused by the RNP complex, yet it might be attributed to inherent genetic variation within the zygotes rather than the outcome of gene editing. Nevertheless, the 3-base deletion of “GTG” and the single-base insertion of “A” are more likely to be attributed to the Cas9-sgRNA complex. Moreover, due to gene locus selection, we were unable to detect visible phenotypic differences early in embryonic development. Meanwhile, the in vitro development of zygotes in the long term was not well established. Only some of the surviving embryos developed into larvae, and we chose to extract the genomic DNA from the larvae; we were unable to validate the phenotypic screening and genome sequencing for adult crabs. In the future, we will test different gene loci and select genes with early phenotypic differences, such as *Hox* gene [22] and *Pax* gene [54].

Zygotes experienced a challenging developmental process, with control hatching rates of ~30% and experimental groups showing very low survival (~0.08%). Electroporation-induced damage was unavoidable, but further optimization of culture methods may improve survival. Editing outcomes were observed in a small number of surviving larvae: 3/4 in the Cas9+sgRNA1 group had indels, and all survivors in the sgRNA2+Cas9+Donor group carried putative insertions. Sanger sequencing confirmed these events, although mosaicism could not be assessed due to limited numbers. These results demonstrate the feasibility of Cas9-based editing in *P. trituberculatus* while highlighting the need to improve survival and culture conditions.

## 5. Conclusions

In this study, we developed a high-throughput, easy-to-operate gene editing method for *P. trituberculatus* using electroporation technology. After preparing the editing components and optimizing the electroporation conditions, we successfully delivered RNP into single-cell zygotes. Mutant individuals were identified under the optimized conditions of 600 V, 1 μF, and two pulses. Using this approach, we detected mutations in the crab larvae, including indels and precise insertions. This study provides a valuable technical reference for exploring gene editing in other non-model crustacean species. In future work, we aim to further optimize target gene selection, in vitro culture conditions, and delivery methods to establish a more efficient gene editing platform for *P. trituberculatus*, advancing precision breeding efforts.

## Figures and Tables

**Table 1:** Plasmids and bacterial strains used in this study.

Strain or Plasmid	Characteristics	Source
Plasmids		
pUC57	Empty plasmid with multiple cloning sites	Lab stock
PMJ922	Cas9 expression plasmid	(Burger et al., 2016) [31]
pDonor-CMV-mcherry	*MSTN* donor and mcherry marker plasmid	This study
pgRNA	sgRNA expression cassette	(Zhou et al., 2020) [32]
Strains		
*E. coli* DH5α	Wild type	
*E. coli* HT115	Wild type	
pUC57/*E. coli* DH5α	Engineering strain containing pUC57 plasmid	Lab stock
pgRNA/*E. coli* DH5α	SgRNA expression	(Zhou et al., 2020) [32]
pDonor-CMV-mcherry/ *E. coli* DH5α	Donor DNA and selective marker of mcherry	This study
PMJ922/*E. coli* HT115	Cas9 expression and purification	(Burger et al., 2016) [31]

**Table 2:** Primer sequence.

	Sequence (5′-3′)	Tm (°C)
M13 F	TGTAAAACGACGGCCAGT	52
M13 R	CAGGAAACAGCTATGACC	52
scaffold F	GTTTTAGAGCTAGAAATAGCAAGT	52
scaffold R	CAGGTCGACGATACTCGAG	52
*mstn* F	GAGCTACCTTGCCCTTTCTGTATTTCCGGC	63
*mstn* R	TCTTTTCCAAATATCTTCCCCCATACATCTATCCCTC	63
sgRNA1 F	GATCACTAATACGACTCACTATAGG**GAAGGTGGTGGTGGTGGTGT**GTTTTAGAGCTAGAAATAGCAAGT	52
SgRNA2 F	GATCACTAATACGACTCACTATAGG**GGACTCCATCACGGTGGTCA**GTTTTAGAGCTAGAAATAGCAAGT	52
sgRNA3 F	GATCACTAATACGACTCACTATAGG**GCAGCAGCTGGTATATCTCT**GTTTTAGAGCTAGAAATAGCAAGT	52
sgRNA4 F	GATCACTAATACGACTCACTATAGG**TTAACCCTTACGAAGGCAAC**GTTTTAGAGCTAGAAATAGCAAGT	52
*MSTN* up F	GTGCC*AAGCTT*GAGCTACCTTGCCCTTTCTGTATTTCCGGCTTCTTGTGGCCTG	72
*MSTN* up R	GCG*TCTAGA*TGCAGGAGTGGCTGACGAGGCCGGAG	72
*MSTN* down F	GCCTTTT*CTCGAG*GTCAGGAGTATAGAAGGAAAGAAGCAG	59
*MSTN* down R	CACCTAA*GGTACC*AGTGCTTTGGTGAGCGTGTGAAT	60
*MSTN* F1	GTCCAGTTGTCTCTGTGGCTTTG	59
*MSTN* F2	GGAAGGACACCAGTAAGGACAACA	59
*MCHERRY* R	CCTTGAAGCGCATGAACTCC	55

Note: Italic fonts with underline represent enzymatic sites; Bold fonts represent 20 nt spacer sequences.

**Table 3:** Development performance of zygotes in 13 d.

Group	Electroporation Parameter	24 h Mortality (%)	96 h Mortality (%)	Surviving Larvae	Larval Ratio (%)	Mutant	Genotype
AS control 1	No pulse	3.46 ± 0.99 ^a^	17.14 ± 2.86 ^a^	723	30.73	\	\
AS control 2	600 V-1 μF-2 pulses	11.09 ± 3.30 ^b^	40.35 ± 5.80 ^b^	23	1.01	\	\
Cas9-eGFP 1	No pulse	3.70 ± 1.52 ^a^	18.26 ± 2.69 ^a^	681	29.12	\	\
Cas9-eGFP 2	600 V-1 μF-2 pulses	16.93 ± 1.05 ^b^	89.21 ± 2.89 ^bc^	3	0.13	\	\
Cas9+sgRNA1	600 V-1 μF-2 pulses	17.19 ± 2.48 ^b^	89.61 ± 7.25 ^bc^	4	0.16	3	Indels
Cas9+sgRNA2	600 V-1 μF-2 pulses	16.14 ± 2.51 ^b^	90.15 ± 2.75 ^bc^	3	0.13	0	\
Cas9+sgRNA1+Donor	600 V-1 μF-2 pulses	20.03 ± 2.66 ^b^	86.67 ± 5.35 ^bc^	2	0.08	0	\
Cas9+sgRNA2+Donor	600 V-1 μF-2 pulses	17.19 ± 2.75 ^b^	91.03 ± 4.84 ^bc^	2	0.08	2	Insertion

Note: Different superscript letters indicate significant differences among groups; “\” represents not determined.

## Data Availability

The data that support the findings of this study are available from the corresponding authors upon reasonable request.

## Supplementary Materials

The following supporting information can be downloaded at https://www.mdpi.com/article/10.3390/molecules31020285/s1, Figure S1: Schematic of pDonor-CMV-mcherry plasmid, showing the upstream and downstream donor sequences flanking the CMV-mCherry cassette; Figure S2: Artificial incubation system for *P. trituberculatus* zygotes used in this study; Figure S3: Comparison of zygotes proportion after Cas9-eGFP electroporation under optimized parameters: 1 μF; voltages of 400, 500, and 600 V; and pulse numbers of 1, 2, and 3. Different superscript letters indicate significant differences among groups; Figure S4: Putative insertion of the CMV-mCherry cassette via CRISPR-Cas9. (A) Schematic diagram of CMV-cassette insertion; (B) Sanger sequencing results of surviving larvae from control and Cas9+sgRNA2+Donor groups. Wild-type sequence from no-pulse group; Mutant1, red capital sequence represents putative insertion of CMV-mcherry cassette. Blue box indicates 20 nt sgRNA sequence; red box indicates 3 nt PAM motif; and orange line indicates PCR and sequencing primers for insertional mutant.

## Abbreviations

The following abbreviations are used in this manuscript:

AS	Artificial Seawater
CRISPR	Clustered Regularly Interspaced Short Palindromic Repeats
DSBs	DNA Double-strand Breaks
HR	Homologous Recombination
LB	Luria–Bertani Broth
mstn	Myostatin gene
NHEJ	Non-Homologous End Joining
PBS	Phosphate-Buffered Saline
PCR	Polymerase Chain Reaction
RNP	Ribonucleoprotein
sgRNA	Single-Guide RNA
