# Supplementary figures and images for: Establishment of CRISPR-Cas9-Mediated Gene Editing in the Swimming Crab Portunus trituberculatus
Source: Molecules. 2026 Jan 13;31(2):285. doi: 10.3390/molecules31020285 (PMC12844060; doi:10.3390/molecules31020285)

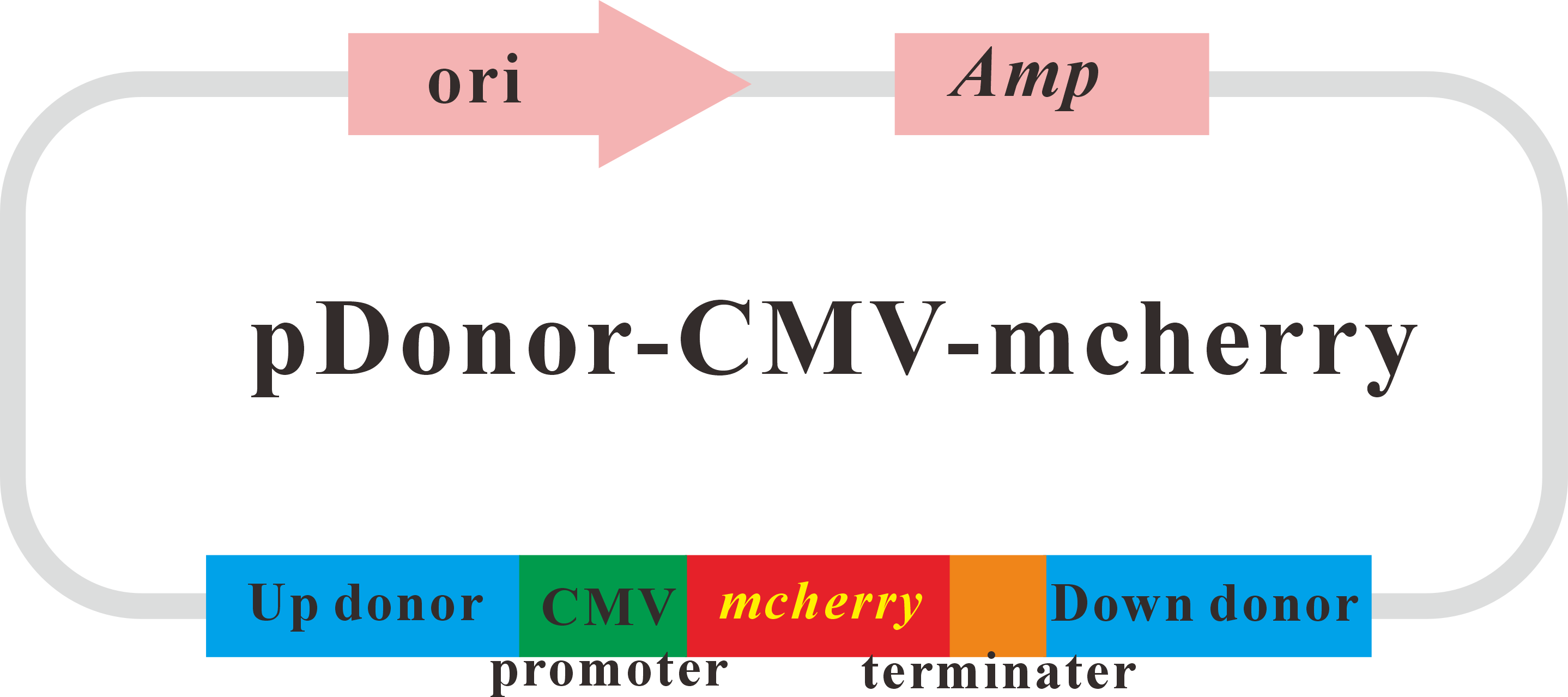

Supplement: Supplementary file 1 [file molecules-31-00285-s001.zip › Figure S1.tif]

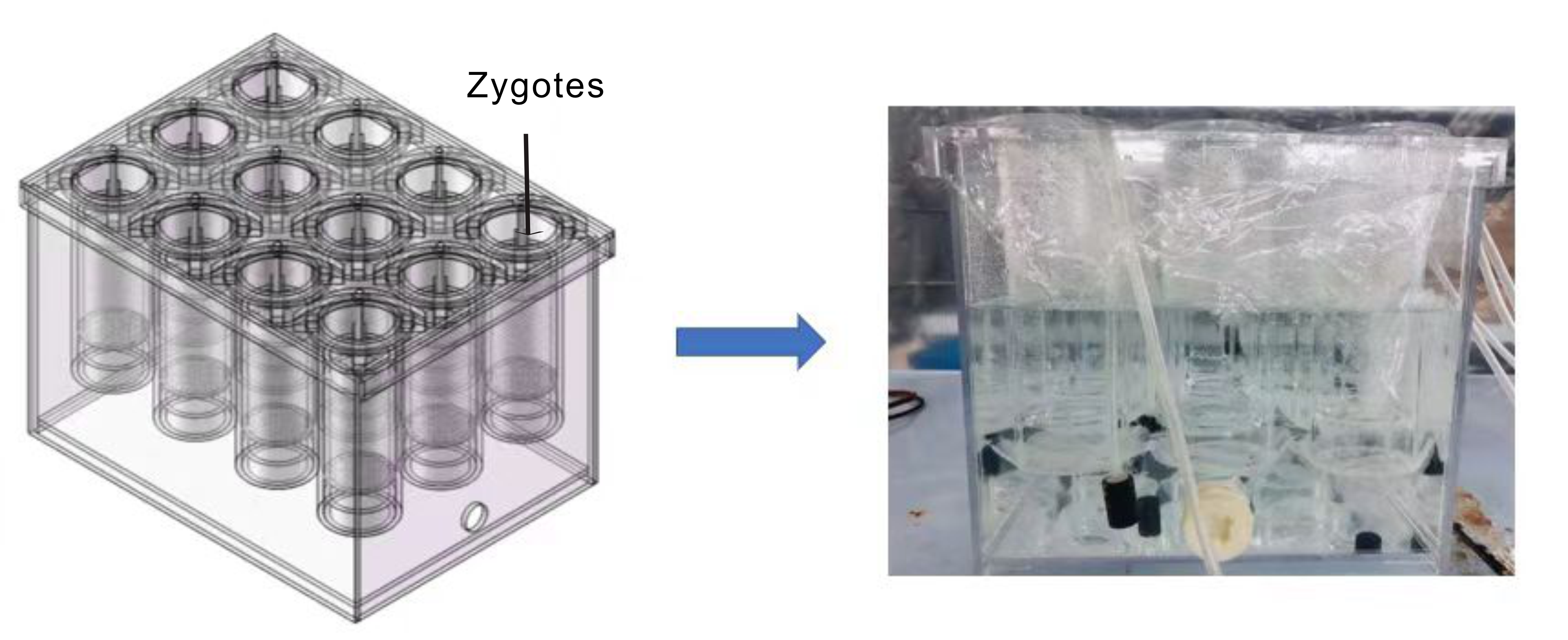

Supplement: Supplementary file 1 [file molecules-31-00285-s001.zip › Figure S2.tif]

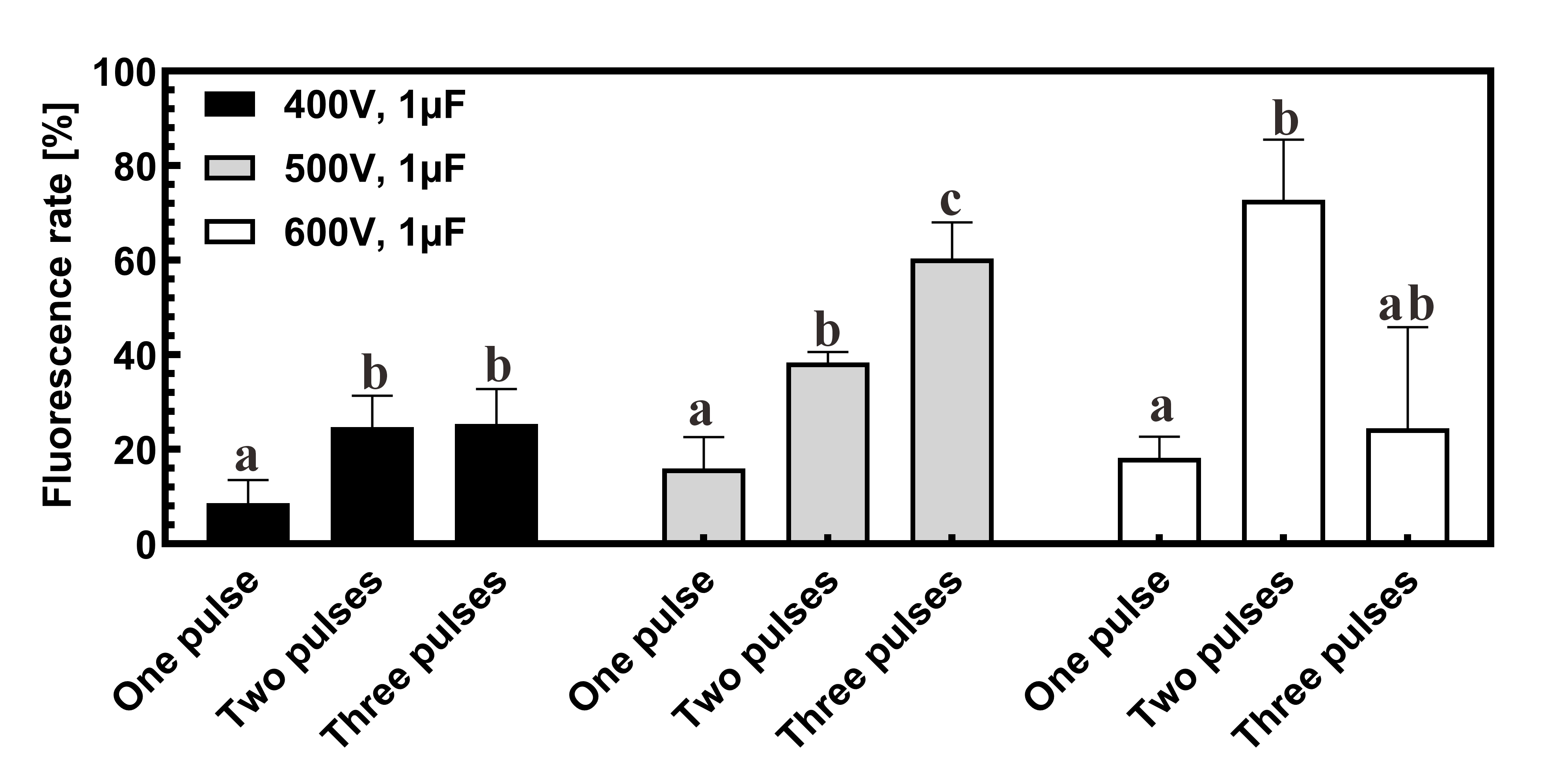

Supplement: Supplementary file 1 [file molecules-31-00285-s001.zip › Figure S3.tif]

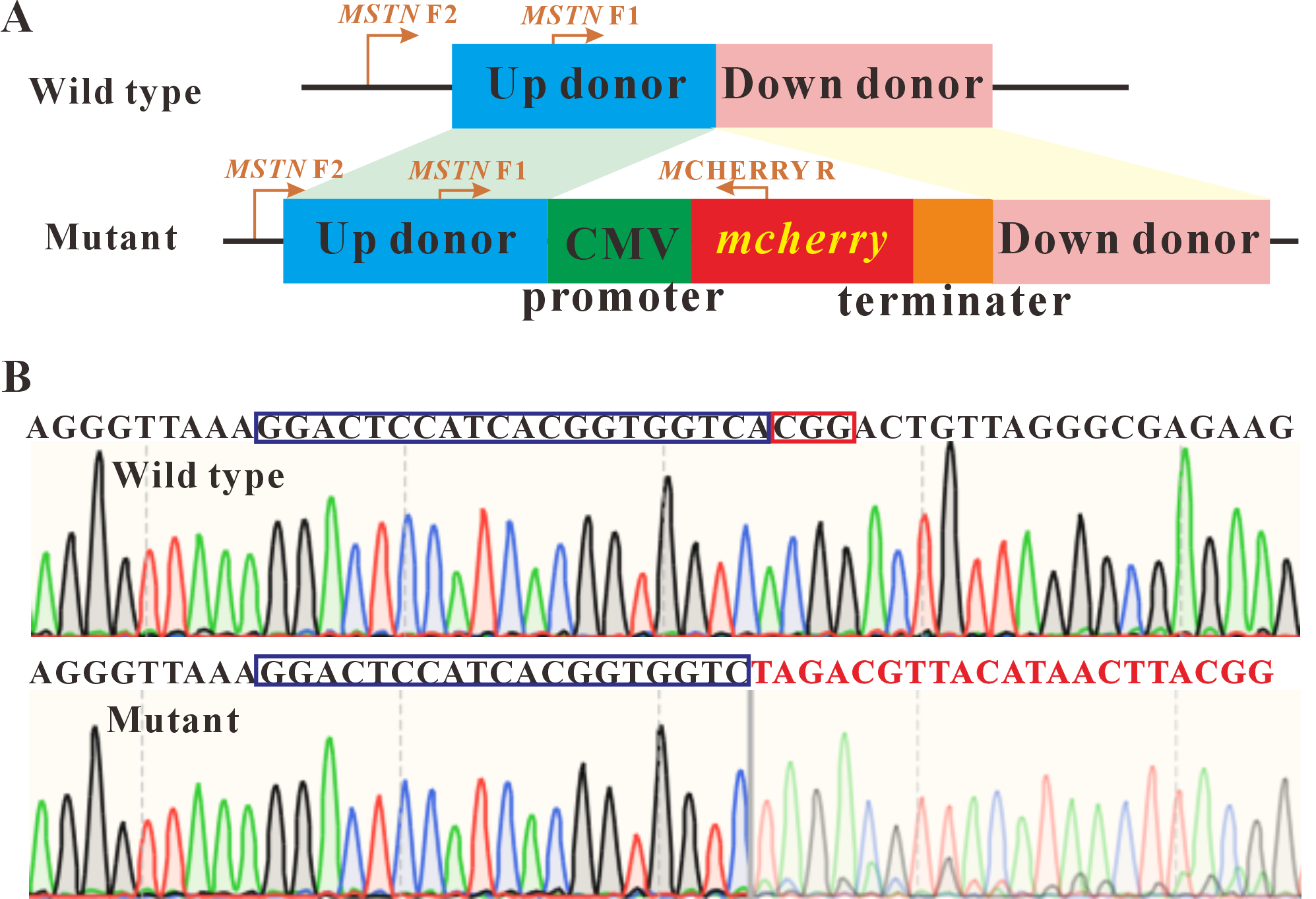

Supplement: Supplementary file 1 [file molecules-31-00285-s001.zip › Figure S4.tif]
